# Age-related decrease in motor cortical inhibition during standing under different sensory conditions

**DOI:** 10.3389/fnagi.2014.00126

**Published:** 2014-06-12

**Authors:** Selma Papegaaij, Wolfgang Taube, Margot Hogenhout, Stéphane Baudry, Tibor Hortobágyi

**Affiliations:** ^1^Center for Human Movement Sciences, University of Groningen, University Medical Center GroningenGroningen, Netherlands; ^2^Movement and Sports Science, Department of Medicine, University of FribourgFribourg, Switzerland; ^3^Laboratory of Applied Biology, Faculty for Motor Sciences, Université Libre de BruxellesBrussels, Belgium; ^4^Faculty of Health and Life Sciences, Northumbria UniversityNewcastle Upon Tyne, UK

**Keywords:** balance, aging, brain aging, transcranial magnetic stimulation, short-interval intracortical inhibition

## Abstract

**Background:** Although recent studies point to the involvement of the primary motor cortex in postural control, it is unknown if age-related deterioration of postural control is associated with changes in motor cortical circuits. We examined the interaction between age and sensory condition in the excitability of intracortical motor pathways as indexed by short-interval intracortical inhibition (SICI) and intracortical facilitation (ICF) during standing.

**Methods:** We used magnetic brain stimulation to evoke SICI and ICF in 11 young (range 21–25 years) and 12 healthy old adults (range 60-74 years) while they stood on a rigid platform or foam, with the eyes open or closed.

**Results:** There was an overall age-related 43% reduction in SICI (*p* = 0.001). SICI lessened when standing on foam in old (31%) but not in young (1%) adults (condition × group interaction, *p* = 0.049). This reduction was associated with increases in center of pressure velocity (*r* = -0.648, *p* = 0.043). Age (*p* = 0.527) and sensory conditions (*p* = 0.325) did not affect ICF.

**Conclusion:** Motor cortical circuits controlling leg muscles are modulated differently in healthy old vs. young adults during upright posture. Future experiments will clarify whether this difference mediates impaired postural control or serves as a compensatory mechanism to counteract postural instability.

## INTRODUCTION

Accumulating evidence points to the involvement of the primary motor cortex (M1) in postural control (Beloozerova et al., 2003; Taube et al., 2006, 2008; Tokuno et al., 2009). Several studies also suggest that training of balance skills is associated with M1 plasticity (Beck et al., 2007; Schubert et al., 2008) and that this plasticity correlates with improvements in postural control (Taube et al., 2007; Taubert et al., 2010). It is well known that postural control of upright stance progressively declines with age. Many factors have been identified to contribute to the age-related deterioration of postural control, including reduced muscle strength (Boelens et al., 2013), impaired sensory abilities (Proske and Gandevia, 2012), slowed nerve conduction velocity (Nardone et al., 1995), and altered spinal reflexes (Koceja et al., 1995; Baudry and Duchateau, 2012). In addition, the excitability of the corticospinal pathway, including the direct projections (monosynaptic) from cortical neurons to spinal motor neurons, is greater in old compared with young adults during upright standing (Baudry et al., 2014a,b). It is unknown if, in addition to age-related changes in the corticospinal pathway, aging also influences intracortical circuits during upright standing.

During manual motor tasks, neuroimaging studies report greater activation in M1, premotor, and prefrontal areas in old compared with young adults (Calautti et al., 2001; Mattay et al., 2002; Ward and Frackowiak, 2003). Similarly, studies using transcranial magnetic stimulation (TMS) revealed disinhibition in several M1 inhibitory circuits in old compared with young adults during manual motor tasks, as reflected by decreases in interhemispheric inhibition, silent period, cortical reciprocal inhibition, and short-interval intracortical inhibition (Oliviero et al., 2006; Hortobagyi et al., 2006; Talelli et al., 2008; Marneweck et al., 2011; Fujiyama et al., 2012; Heise et al., 2013). However, several other studies report no changes (Oliviero et al., 2006; Smith et al., 2011) or even increased inhibition with age (Kossev et al., 2002; McGinley et al., 2010). These inconsistencies in literature are probably related to differences in task difficulty, age range and the type of muscle examined. Age-related decreases in intracortical inhibition have been associated with worse motor performance measured under a variety of experimental conditions (Marneweck et al., 2011; Fujiyama et al., 2012; Heise et al., 2013). Thus, there is ample evidence that aging modifies intracortical processing and that such modifications can affect motor control (for a review, see Papegaaij et al., 2014). Hence, it seems reasonable to expect that age-related changes in intracortical circuits are – at least in part – responsible for the deterioration in postural control.

Therefore, the purpose of the present study was to examine the interaction between age and sensory condition in M1 excitability as indexed by short-interval intracortical inhibition (SICI) and intracortical facilitation (ICF) during standing. We manipulated sensory condition by altering proprioceptive feedback (standing on a rigid platform vs. foam) and visual feedback (eyes open vs. eyes closed).

Based on work showing modulations in inhibitory spinal circuits with sensory conditions and postural task difficulty (Koceja et al., 1995; Katz et al., 1988; Baudry and Duchateau, 2012), we hypothesized that behaviorally relevant modulations would also occur in M1 excitability. Specifically, we expected a reduction of SICI with altered sensory conditions, to increase M1 excitability and consequently the number of motor solutions available. In addition, based on the previously mentioned studies reporting cortical disinhibition with aging (Oliviero et al., 2006; Hortobagyi et al., 2006; Talelli et al., 2008; Marneweck et al., 2011; Fujiyama et al., 2012; Heise et al., 2013), we expected that old adults would show a generally reduced SICI compared to young adults. Based on the findings of Heise et al. (2013), we hypothesized that this age-related disinhibition during normal standing would result in a decreased modulation of SICI between tasks. To validate the putative role of M1 in postural control, we predicted an association between the task-related modulation of M1 excitability and alteration in postural stability. Because of a lack of data concerning ICF modulation during postural tasks, the formulation of a specific hypothesis is premature.

## MATERIALS AND METHODS

### PARTICIPANTS

Eleven healthy young adults (age 23 ± 1 years, range 21–25 years, 4 men) and fourteen healthy old adults (age 68 ± 5 years, range 60–77 years, 11 men) volunteered for the study. In two old men (age 66 and 77 years) the stimulation intensity was above the comfort threshold and we stopped data collection. Two young and two old adults were left-footed (Hebbal and Mysorekar, 2006). None of the participants had a history of or presented with neurological disorders, severe orthopedic disorders, suspicion of pregnancy, non-dental associated metal within the cranium, or took neuroactive drugs or drugs known to affect balance. The mini-mental state examination (MMSE) and short questionnaire to assess health-enhancing physical activity (SQUASH) were used to determine general cognitive function and physical activity in daily life. Subjects also completed the short physical performance battery (SPPB) including standing balance, walking speed and chair stand tests to specifically evaluate lower extremity function (**Table 1**). Before the experiment, subjects signed an informed consent document approved by the Medical Ethics Committee of the University Medical Center Groningen.

**Table 1:** Subject characteristics.

	Young adults	Old adults
Age (years)	23 ± 1	68 ± 4
Sex (male; female)	4; 7	11; 3
BMI (kg/m^2^)	23 ± 1.7	24 ± 3.6
SPPB score	12 ± 0	12 ± 1
MMSE score	30 ± 0	30 ± 1
**SQUASH**
Total score	9718 ± 1934	10045 ± 3236
Light (min/w)	2337 ± 580	912 ± 829
Moderate (min/w)	459 ± 309	353 ± 284
Heavy (min/w)	222 ± 229	602 ± 218

*Values are mean ± SD, unless indicated differently. BMI: body mass index, SPPB: short physical performance battery (max. score of 12), MMSE: mini mental state examination (max. score of 30), SQUASH: short questionnaire to assess health-enhancing physical activity. Total score is minutes per week × intensity of the activity. The amount of light, moderate and heavy exercise is expressed in minutes per week.*

### EXPERIMENTAL SETUP

Surface electromyography (EMG) was recorded (DE-2.1, Delsys, Natick, MA, USA) for the right tibialis anterior (TA) by attaching active electrodes over the muscle belly and the reference electrode on the medial aspect of the tibia. To minimize impedance at the electrode-skin contact, the skin was shaven, abraded with fine-grain sandpaper, and cleaned with alcohol. The EMG signal was amplified 1000 times (model Bagnoli-8, Delsys, Natick, MA, USA), sampled at 5 kHz, and bandpass filtered with a second order Butterworth filter (10–1000 Hz) using data acquisition interface and software (Power 1401 and Spike2, Cambridge Electronics Design, Cambridge, UK). Subjects performed a maximum voluntary contraction (MVC) of the ankle dorsal flexors while seated in a chair with the knee in 45° flexion and the ankle in neutral position. The EMG activity recorded during this effort was used to express and normalize the background EMG.

During the main part of the experiment, subjects were instructed to maintain an upright bipedal stance on two force plates (Bertec 4060-08, Columbus, OH, USA). With the arms crossed across the chest, subjects looked at a sharply visible “+” sign displayed on a projection screen. Foot position was standardized with the heels 9 cm apart and a toe-out angle of 30°. The center of pressure (CoP) position signal was sampled at 100 Hz, and filtered using a fourth order low-pass Butterworth filter with a cut off frequency of 10 Hz. It was not necessary to correct the CoP position data for height of the foam (6 cm) because pilot experiments showed a minimal effect.

An 11-video-camera motion analysis system (Vicon, Oxford, UK) recorded spatial coordinates of reflective markers placed on the right trochanter major and lateral malleolus. The signal was sampled at 100 Hz, filtered with a second order low-pass Butterworth filter (cut off frequency: 5 Hz), and used online to determine whether the person was swaying forward or backward.

In a random order, subjects performed four standing conditions with altered sensory states: standing on a rigid surface with eyes open (rigid – EO) and eyes closed (rigid – EC), standing on foam (47 cm × 38 cm × 6 cm, Bodybow Healthcare BV, Nieuwegein, Netherlands) with eyes open (foam – EO) and eyes closed (foam – EC). Three minute resting periods were given between conditions to prevent fatigue. We also recorded two periods of 10 s per surface condition (rigid, foam) where participants assumed the standardized position without receiving TMS.

### TMS DATA ACQUISITION

In the present study, transcranial magnetic stimuli were delivered over the left M1 with a double cone coil connected to a Magstim 200^2^ and Bistim^2^ (Magstim, Whitland, UK), except for two old adults for whom a 90 mm circular coil was used as no clear MEP was evoked with the double-cone coil. We targeted the TA because this muscle generally provides a low motor threshold (MT) and good reliability of MEPs (Cacchio et al., 2009). The optimal location for eliciting MEP’s in the TA with the largest amplitude at a given intensity was determined by moving the coil systematically in steps of 0.5 cm over the M1 area starting at the vertex. In general, the optimal location was found 0.5–1.5 cm posterior and lateral to the vertex. The location was marked on the skull with a permanent marker to enable the experimenter to hold the coil on a consistent location throughout the experiment. While standing, the MT, defined as the lowest intensity in which the MEP’s were larger than 100 μV in at least three out of five consecutive trials, was determined (Calautti et al., 2001).

Paired-pulse TMS with an interstimulus interval of 2.5 ms was used to assess SICI, while an interstimulus interval of 13 ms was used to assess ICF. The interstimulus intervals were chosen based on the literature (Roshan et al., 2003; Soto et al., 2006) and pilot experiments showing greatest inhibition and facilitation at these intervals. Conditioning and test stimulation intensity were set at 0.8 and 1.2 MT, respectively. In all standing conditions there were 10 test MEP, 10 SICI, and 10 ICF trials. To reduce variability in MEP size induced by sway direction (Tokuno et al., 2009) and to minimize the magnitude of background EMG, TMS was triggered only when the person was swaying forward (negative angular velocity) and with a minimal interval of 5 s between trials.

### DATA ANALYSIS

Rectified background EMG was averaged in each trial over the 100-ms period preceding the TMS artifact, and expressed as a percentage of the EMG activity measured during the MVC trial. The peak-to-peak amplitude of the TMS-generated MEPs was computed. Using the interquartile range (Tukey, 1977), four percent of the total number of trials were identified as outliers and were substituted with the mean. SICI and ICF were expressed as percentage inhibition and facilitation, by using the following formula for SICI: 100 - (conditioned MEP/test MEP × 100), and the following formula for ICF: (conditioned MEP/test MEP × 100) - 100. CoP velocity while standing on the rigid platform and on foam was used to describe the postural behavior of subjects. CoP velocity is a highly reliable measure (Demura et al., 2008) that has proven to discriminate well between age groups and test situations (Raymakers et al., 2005).

### STATISTICAL ANALYSES

**FIGURE 1:**
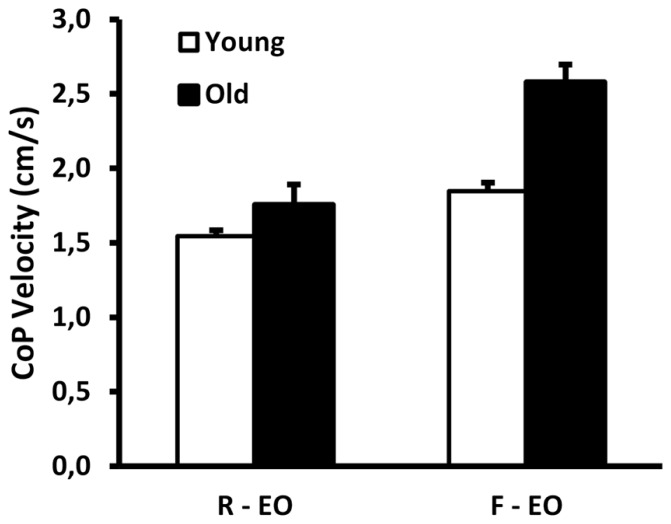
**Group data (mean ± SE) for young and old adults of center of pressure velocity when standing on a rigid (R) surface and on foam (F), showing a significant interaction effect (*p* = 0.001) EO: eyes open**.

All variables were checked for normal distribution prior to analysis. Background EMG was logarithmically transformed because of a skewed distribution. We compared MT between young and old adults using an independent samples *t*-test. The main analysis was an Age (young, old) by Condition (rigid-EO, rigid-EC, foam-EO, foam-EC) two-way repeated measures ANOVA on test MEP amplitude, SICI, ICF, and background EMG. In case of a significant condition effect, results were subjected to a *post hoc* Tukey’s test. Because CoP velocity was only measured in two conditions (rigid, foam), this outcome measure was analyzed in an Age (young, old) by Condition (rigid, foam) two-way repeated measures ANOVA. A Greenhouse–Geisser correction was applied when the assumption of sphericity was violated. Pearson correlation coefficients were computed to assess the association of background EMG with test MEP amplitude, SICI and ICF, and the association of test MEP amplitude with SICI, using the combined age group data. Furthermore, Pearson correlation coefficients were computed to examine the relationship between the changes in SICI and CoP velocity from rigid to foam conditions in the two age groups. IBM SPSS statistics 20 was used for statistical analysis. The alpha level was set at 0.05. Results are presented as mean ± SD in the text and tables and mean ± SE in **Figures 1** and **3**. Interaction effects are only reported when significant.

## RESULTS

### CENTER OF PRESSURE

There was a significant age × condition interaction effect for CoP velocity (*F*_1,19_ = 15.8; *p* = 0.001; **Figure 1**), with a greater increase in CoP velocity from the rigid to the foam condition in old adults (from 1.75 to 2.58 cm/s, *p* < 0.001) as compared to young adults (from 1.54 to 1.85 cm/s, *p* < 0.001).

### TMS MEASURES

**FIGURE 2:**
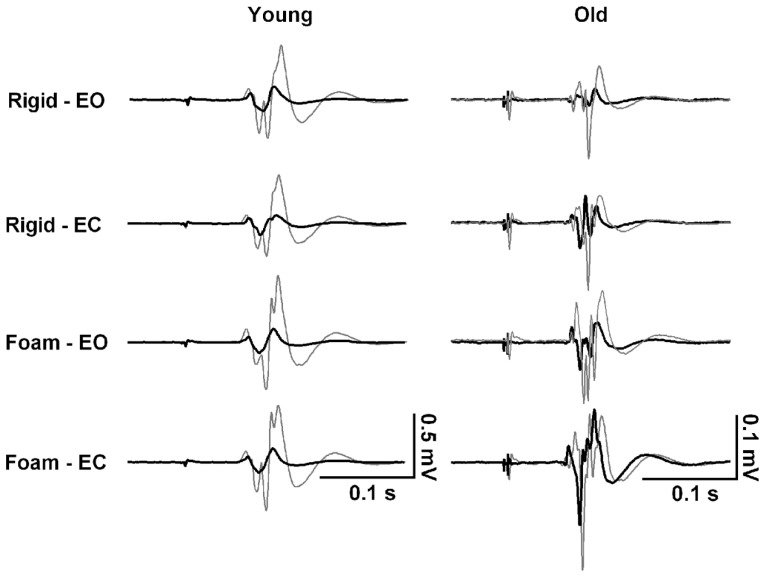
**Representative responses to transcranial magnetic brain stimulation in the tibialis anterior muscle of one 23-year-old female and one 73-year-old male participant while standing on rigid and foam surfaces with eyes open (EO) and eyes closed (EC).** Waveforms represent the motor evoked potential averaged over 10 trials in response to an unconditioned test pulse (thing gray line) and to a conditioned test pulse (thick black line) at 2.5 ms interval. Note the absence of modulation of short interval intracortical inhibition (SICI) across the four conditions in the young subject, in contrast to the old subject who decreases SICI when sensory conditions are altered.

**FIGURE 3:**
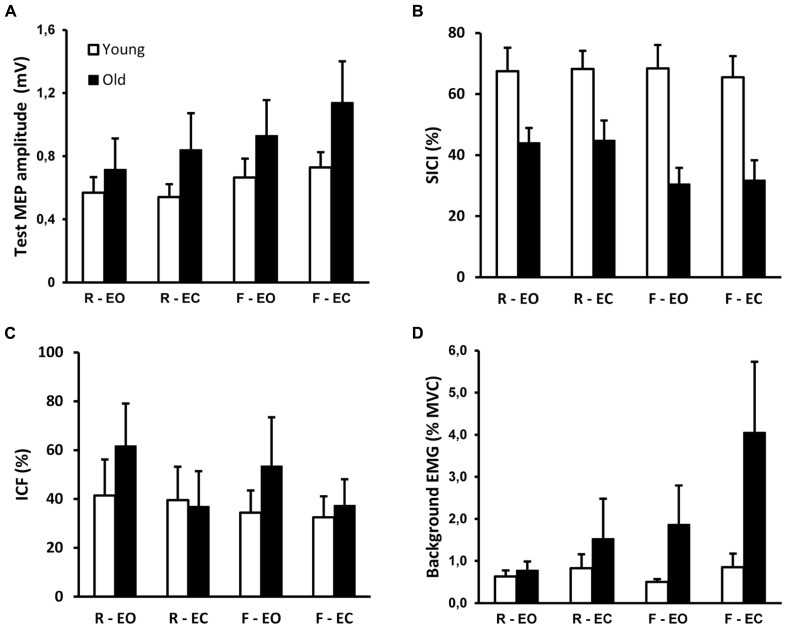
**Group data (mean ± SE) for young and old adults of (A) amplitude test MEP (condition effect, *p* < 0.001), (B) short interval intracortical inhibition (SICI; group effect, *p* = 0.001; interaction effect, *p* = 0.049), (C) intracortical facilitation (ICF), (D) background EMG (interaction effect, *p* = 0.031).** Conditions were standing on a rigid platform and on foam (rigid, foam), with eyes open and eyes closed (EO, EC). Greater values for SICI and ICF represent respectively more inhibition and facilitation.

Across all subjects, MT was similar in young (49 ± 8%, range 34–55%) and old adults (50 ± 12%, range 31–73%, *t*_21_ = -0.2, *p* = 0.845). Therefore, the average stimulation intensities were similar in the two age groups. The effects of sensory condition on TMS responses from a representative young and old subject are illustrated in **Figure 2**. Group data show that test MEP amplitude was similar in young and old adults (*F*_1,20_ = 1.3, *p* = 0.261; **Figure 3A**). However, there was a significant condition effect (*F*_2,39_ = 14.2, *p* < 0.001). *Post hoc* tests revealed that the test MEP’s during standing on foam were greater compared with those recorded while standing on a rigid surface (*p* < 0.01), and during foam-EC compared with foam-EO (*p* < 0.001). However, no difference related to vision conditions was observed when standing on a rigid surface (*p* > 0.05). The 2 × 4 ANOVA did not reveal significant modulation between conditions for SICI (*F*_3,60_ = 1.7, *p* = 0.185), but SICI was greater in young adults (67 ± 23%) compared to old adults (39 ± 20%) regardless of standing condition (*F*_1,20_ = 16.5, *p* = 0.001; **Figure 3B**). Furthermore, when pooled across vision conditions, the 2 (young, old) by 2 (rigid, foam) ANOVA revealed a significant age × condition interaction (*F*_1,20_ = 4.4, *p* = 0.049), with a decrease in SICI from rigid to foam in old (31% change, *p* < 0.001) but not in young adults (*p* > 0.05). Age and conditions did not affect ICF (*F*_1,20_ = 0.4, *p* = 0.527; *F*_3,60_ = 1.2, *p* = 0.325; **Figure 3C**).

### BACKGROUND EMG

**Figure 3D** shows the group × condition interaction (*F*_2,34_ = 4.1; *p* = 0.031) for the log-transformed background EMG in the TA, with a greater increase in background EMG when altering the sensory conditions in old compared to young adults. *Post hoc* tests revealed that there was no effect of sensory condition on background EMG in young adults (*p* > 0.05). In old adults all comparisons between conditions were significant (*p* < 0.05), except for rigid-EO vs. rigid-EC (*p* > 0.05). Moreover, bEMG did not differ between age groups during the rigid surface conditions (*p* > 0.05), but was higher in old adults during the foam surface conditions (*p* < 0.001).

### CORRELATIONS

To determine whether differences in background EMG could have caused the condition (rigid vs. foam) and age (young vs. old) effects in TMS measures, we performed correlation analyses. The changes in background EMG between the rigid and the foam conditions did not correlate significantly with the changes in test MEP amplitude (*r* = 0.27, *p* = 0.244), SICI (*r* = -0.29, *p* = 0.202) or ICF (*r* = -0.25, *p* = 0.275). Furthermore, there was no significant correlation between the changes in test MEP amplitude and SICI (*r* = -0.26, *p* = 0.239) or ICF (*r* = -0.17, *p* = 0.444).

**FIGURE 4:**
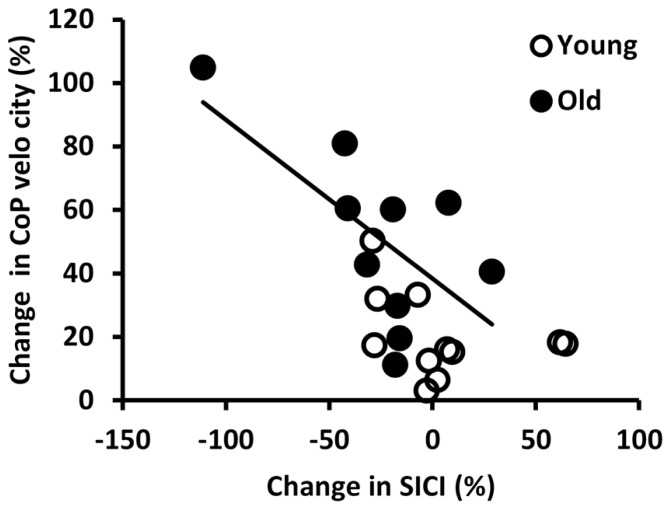
**Correlation between the percent changes in short interval intracortical inhibition (SICI) and center of pressure (CoP) velocity from the rigid to the foam condition in young and old adults.** The regression line illustrates that in old subjects greater reductions in SICI were accompanied by greater increases in CoP velocity (*r* = -0.648, *p* = 0.043).

**Figure 4** shows the relationship between changes in SICI and CoP velocity from the rigid to the foam condition in young and old adults. Within the old adults, subjects who reduced their SICI more when changing from standing on a rigid surface to standing on the foam, showed a greater increase in CoP velocity (*r* = -0.65, *p* = 0.043).

## DISCUSSION

This is the first study that examines the effects of age on intracortical circuit excitability during postural tasks. The data confirmed the overriding hypothesis of an interaction between age and sensory condition in M1 excitability: SICI lessened when standing on foam compared with standing on a rigid surface in old but not in young adults. This reduction was associated with a greater decrease in stability when the support surface was altered. In contrast, ICF did not vary with age and sensory conditions. This study extends the literature reporting on the age-related reductions in cortical inhibition during manual tasks by demonstrating similar reductions in cortical inhibition during a postural task.

### AGE-RELATED DECREASE IN SICI

Previous studies reported inconsistent results concerning the association between age and SICI. The inconsistencies may in part be due to differences in the strength of corticomotoneuronal projections to the target muscle. For example, hand muscles compared with forearm muscles have more abundant monosynaptic projections (Palmer and Ashby, 1992), and SICI investigated in motor cortex associated with hand muscles has been reported to be reduced (Marneweck et al., 2011; Heise et al., 2013) or similar (Oliviero et al., 2006; Smith et al., 2011) in old compared with young adults. In contrast, studies examining wrist flexors and extensors found more SICI in old than in young adults (Kossev et al., 2002; McGinley et al., 2010). As the TA exhibits nearly similar density of corticospinal projections as hand muscles (Brouwer and Ashby, 1992; Petersen et al., 2003), results of the present study support the hypothesis of a specific reduction in SICI for muscles with rich corticospinal projections, suggesting that aging further strengthens the corticomotoneuronal pathway by diminishing intracortical inhibition. Nonetheless, this may also reflect a greater contribution of this pathway during upright standing, regardless of leg muscles (Baudry et al., 2014b).

### POSTURE-SPECIFIC REDUCTIONS IN SICI AFFECT PERFORMANCE

In addition to the lower SICI in elderly adults, the present results show that elderly adults modulated SICI depending on the sensory conditions, and more specifically the surface of support. There are several studies showing changes in SICI related to motor control. For example, SICI is reduced during movement preparation (Heise et al., 2013), during the activation phase compared with the deactivation phase in cycling (Sidhu et al., 2013), and after periods of motor practice (Rosenkranz et al., 2007; Smyth et al., 2010). However, we found no difference in SICI between tasks in young adults, resulting in a significant age × condition interaction. It must be noted that this interaction was only significant when data was pooled across vision conditions, probably due to a relatively low number of participants. Also, this might indicate that the modulation only occurs when balance is truly challenged, as changing the surface usually has a bigger impact than removing vision (Baudry and Duchateau, 2012). The lack of modulation in SICI in young adults is in line with a previous study that reported similar SICI levels during voluntary and postural contraction of the soleus muscle, suggesting that SICI is not specifically modulated during upright standing in young adults (Soto et al., 2006). This may be because increasing M1 excitability would not improve muscle coordination needed to perform a difficult postural task. It may also be that the postural tasks were not challenging enough for the young adults to require changes in neural control, although young adults did increase CoP velocity when standing on foam.

Nonetheless, old adults exhibited a lower level of SICI when standing on foam compared to standing on a rigid surface. Within the group of old adults, reductions in SICI correlated with increases in CoP velocity. This is consistent with other studies reporting a correlation between low intracortical inhibition and poor motor performance in old adults (Marneweck et al., 2011; Fujiyama et al., 2012; Heise et al., 2013). Lower SICI was associated with poorer performance in the Purdue Pegboard test for manual dexterity, but not with performance on a pinch grip force control task (Marneweck et al., 2011). The correlation was driven by a number of (primarily old) subjects who exhibited atypically low inhibition, appearing as facilitation. There was also an association between the strength of intracortical inhibition measured by the contralateral silent period during an interlimb coordination task and the performance on this task (Fujiyama et al., 2012). At last, there was a link between the level of SICI at rest and single reaction time and two finger tapping speed (Heise et al., 2013). Although all three papers (Marneweck et al., 2011; Fujiyama et al., 2012; Heise et al., 2013) found an association between age-related disinhibition and reductions in motor performance, these studies did not quantify changes between tasks, instead reported inhibition only during one specific task (Marneweck et al., 2011; Fujiyama et al., 2012) or at rest (Heise et al., 2013). Therefore, the present study is the first to show that task-related modulation in SICI can be linked to behavior in old adults. One interpretation could be that the task-specific reduction in SICI was dysfunctional, negatively affecting postural control. As phasic activation of GABA-A receptors is thought to contribute to the regulation of synchronous motor neuronal activity (Farrant and Nusser, 2005), it is suggested that a general disinhibition could interfere with this process (Heise et al., 2013), and therefore induce difficulties when one is required to coordinate the fast contractions of different muscles during standing on foam.

However, we cannot exclude the possibility that the reduction in SICI in old adults was a compensatory mechanism. It can be argued that standing on foam is a relatively more difficult task for old than young subjects, and that the more subjects were challenged the more they were ‘forced’ to facilitate M1 activity. Therefore, neither the previous nor the current data can determine whether increased instability caused reduced SICI or the other way around.

### NO AGE-RELATED CHANGES IN ICF

We did not find differences in ICF between young and old adults, which is consistent with two studies that examined ICF in the first dorsal interosseus muscle (Smith et al., 2009, 2011). However, it is inconsistent with two other studies that reported lower ICF in the wrist flexors and extensors of old compared with young adults (Kossev et al., 2002; McGinley et al., 2010). This is in line with the earlier proposed shift towards an age-related facilitation of the muscles with strong corticospinal projections. Given the decrease in SICI, the lack of change in ICF may indicate that modulation of cortical circuits with sensory conditions relies more on reducing strength of inhibitory inputs to increase the potential for more cortical contribution to control leg muscles rather than increasing cortical activity. This is in agreement with the lack of changes in test MEP observed in the present study and the absence of modulation in corticomotoneural excitability with altered proprioception (Baudry et al., 2014b). Such adjustments may be relevant to increase the cortical contribution to postural control without increasing cortical activity that may induce noisy neural signal.

Although many studies have used ICF to investigate cortical excitability (Ginanneschi et al., 2005; Soto et al., 2006; Smyth et al., 2010; Howatson et al., 2011), the neurophysiological origin of ICF is still under debate (Reis et al., 2008). As N-methyl-D-aspartate (NMDA) receptor antagonists and glutamate antagonists decrease ICF, it is often believed to reflect glutaminergic intracortical circuits (Liepert et al., 1997; Ziemann et al., 1998). However, there is some evidence that GABAergic inhibition can modulate ICF (Ziemann et al., 1996). Furthermore, spinal contributions cannot be excluded (Di Lazzaro et al., 2006). Therefore, conclusions regarding ICF should be used with caution, and short interval intracortical facilitation (SICF), an often overlooked measure, might actually be more valid for determining cortical facilitation (Di Lazzaro et al., 1999; Clark et al., 2011).

### THE INFLUENCE OF BACKGROUND EMG

One limitation of this study is the difference in background EMG between conditions and groups. Since the level of muscle contraction could affect SICI, this might have influenced the results. However, there are several reasons why we think this is not the case. First, the contractions were of very low intensity, with an average of 0.9 and 4.1% of MVC in young and old adults during the most difficult condition. Second, the age-related decrease in SICI was also apparent in the rigid-EO condition, while the background EMG did not differ between groups in that condition. Third, the modulation between conditions in SICI did not correlate with the modulation of background EMG.

### FUTURE RECOMMENDATIONS AND CONCLUSIONS

Future studies are needed to better understand the cause-effect relationship between SICI and postural control in aging. One aspect in these studies could be to test the hypothesis of an interaction between age and the strength of corticospinal projection to the target muscle on the amount of SICI and ICF by targeting several muscles. If this proves to be true, such data would address many inconsistencies reported in literature and provide a deeper insight into the age-related changes of the neuromotor system. Another aspect could be the association between behavioral and neurophysiological changes after balance training.

In conclusion, old compared with young adults exhibited an overall decreased level of SICI and reduced this even further when standing with altered proprioception. The reduction in SICI between tasks was associated with an increased velocity of the CoP. This suggests that motor cortical circuits control upright posture differently in old vs. young adults. Future experiments will clarify whether this difference in control mediates impaired postural control or serves as a compensatory mechanism to counteract postural instability.

## Conflict of Interest Statement

The authors declare that the research was conducted in the absence of any commercial or financial relationships that could be construed as a potential conflict of interest.
